# Two-year follow-up of a young male with possible acute hemorrhagic leukoencephalitis: A case report

**DOI:** 10.1097/MD.0000000000032073

**Published:** 2022-12-02

**Authors:** Chen Wu, Weiliang Zhang, Yan Jiao, Minhui Dong, Heng Zhou, Yun Lv, Jun Yang

**Affiliations:** a Department of Neurology, Xi’an Jiaotong University Second Affiliated Hospital, Shaanxi, China; b Department of Neurology, General Hospital of Xinjiang Military Region of the Chinese People’s Liberation Army, Xinjiang, China; c Department of Anesthesia, General Hospital of Xinjiang Military Region of the Chinese People’s Liberation Army, Xinjiang, China.

**Keywords:** acute hemorrhagic leukoencephalitis, follow-up, fulminant demyelinating disease, neuroimage

## Abstract

Acute hemorrhagic leukoencephalitis (AHLE) is a rare but fetal fulminant demyelinating disease of unknown etiology. It is commonly regarded as a severe variant of acute demyelinating encephalomyelitis (ADEM). Its rapid clinical deterioration and high mortality appeal to clinicians to attach importance to early diagnosis. Immunosuppressive therapy is the main management to attenuate the autoimmune process, but with varied response and prognosis.

**Patient concerns:**

A young male presented with moderate fever, headache and seizures after extraction of impacted teeth, and then deteriorated rapidly to deep coma. Initial magnetic resonance imaging (MRI) revealed multiple plaque-like lesions in bilateral cerebra, right thalamus and pontobulbar region with massive edematous swelling and multifocal small hemorrhagic foci. Inflammatory parameters in the peripheral blood were only mild higher with a pleocytosis in CSF.

**Diagnosis:**

His clinical presentation, laboratory evaluation and radiological features were consistent with a suspected diagnosis of AHLE.

**Interventions:**

He underwent pulse corticosteroids initially but failed to respond to it. However, his consciousness improved obviously when he was treated with multiple courses of intravenous injection of immunoglobulin (IVIG) combined with mycophenolate mofetil (MMF).

**Outcomes:**

The patient regained consciousness gradually on day 180 and was in minimally conscious state (MCS) during the two-year follow-up.

**Lessons:**

AHLE presents distinctly from classical ADEM, and the situation may pose a diagnostic challenge. Clinicians should be vigilant in recognizing AHLE because of its rapid clinical deterioration and high mortality. We highlight the critical role of multimodal MRI, particularly susceptibility-weighted imaging (SWI) in the diagnosis of AHLE if cerebral biopsies are unavailable. Multiple courses of IVIG with MMF may be effective when early single pulse of corticosteroids fails. Individual who survives the initial insult may carry relatively good prognosis.

## 1. Introduction

Acute hemorrhagic leukoencephalitis (AHLE) or acute hemorrhagic encephalomyelitis (AHEM), was first described by Weston Hurst in 1941. It is commonly regarded as a rare and severe variant of acute demyelinating encephalomyelitis (ADEM) and was seen in around 2% of ADEM children.^[1,2]^ AHLE is typically characterized by abrupt onset of neurologic deficits and deterioration of consciousness after a preceding infection or vaccination.^[3]^ Death usually occurring within days to 1 week. Multifocal brain lesions with profound edematous necrosis and hemorrhage account for generous poor prognosis with mortality as high as 70%.^[4,5]^ Neurological sequelae remained in surviving patients compared with a complete recovery of 60% to 80% among patients with ADEM.^[6]^ Magnetic resonance imaging (MRI) features of AHLE bear resemblance to encephalitis, fulminant demyelinating diseases, CNS vasculitis, leukoencephalopathies, which makes it difficult to distinguish at the onset.^[7]^ To date, few cases of AHLE in literature have been documented on the Mainland of China. Herein we report a unique presentation of AHLE with long-term follow-up including detailed MRI and electroencephalogram (EEG) profiles.

## 2. Case presentation

A 33-year-old male presented with fatigue, headache and moderate fever (38.6 ℃) two days after catching cold, and then developed generalized tonic‐clonic seizure with remission. The medical history was extraction of two impacted tooth 2 weeks ago. At emergency department, the head CT scan revealed asymmetrical white matter hypodensity in the right frontal lobe and was diagnosed with encephalitis. During the first three days of admission, his symptoms worsened with progressive impairment of consciousness, persistent lower fever and myocardial damage. Thus he was referred to our tertiary medical care center later. Clinical examination revealed a moderate comatose state (GCS score = 7) and pendulum nystagmus, symmetric pupils were reactive to light but oculocephalic and corneal reflexes could not be triggered. The upper deep tendon reflexes existed but the lower deep tendon reflexes were absent. He had no evidence of extensor toe signs and meningeal irritation.

**Figure 1.**
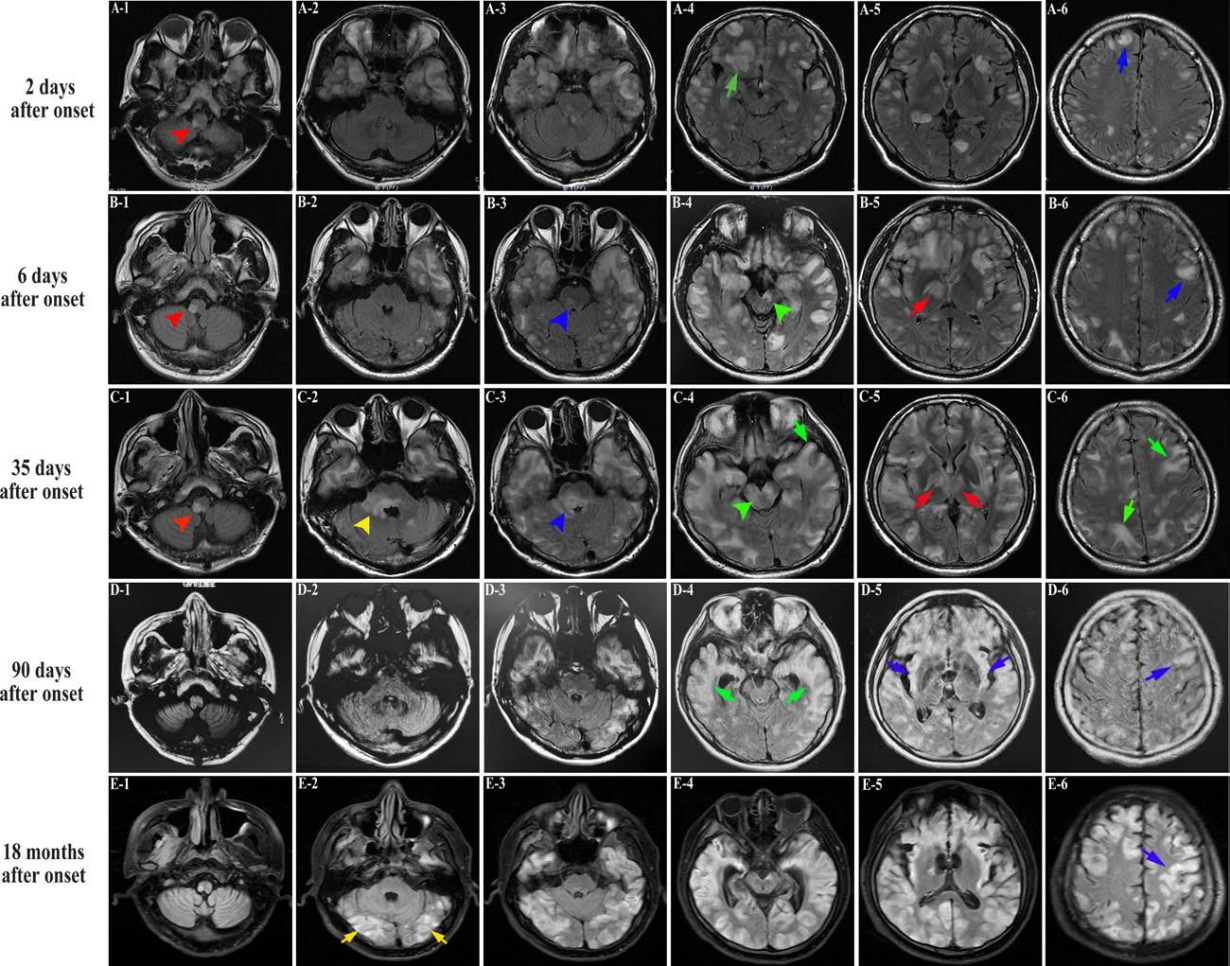
Axial T2-weighted FLAIR images showing lesions in each section at different times after onset. Second day (A1‐A6); 6th day (B1‐B6); 35th day (C1‐C6); 90th day (D1‐D6); 18th months (E1‐E6); confluent widespread hyperintensities of T2-FLAIR sections demonstrate the involvement of medulla (red arrowhead in A1, B1, C1) pons (blue arrowhead in B3, C3) midbrain (green arrowhead in B4, C4), medipeduncle (yellow arrowhead in C2), cerebellar hemisphere (yellow arrow in E2), thalami (red arrow in B5, C5) cortex (blue arrow in A6, B6, D5, D6, E6) subcortex and white matter (green arrow in A4, C4,C6, D4). MR images on day 6 and 35 reveal marked increased number and mass effect of the lesions.

**Figure 2.**
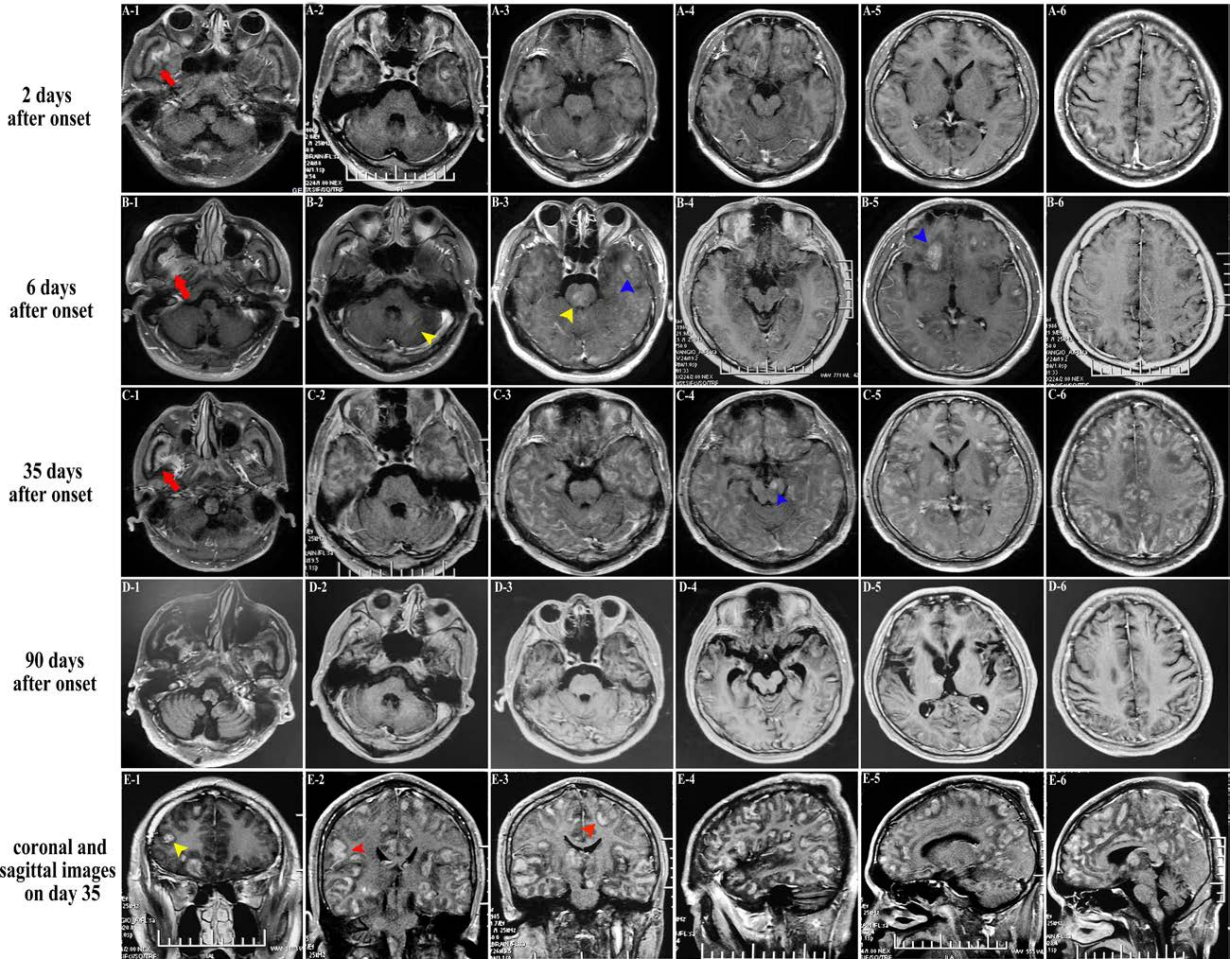
T1-weighted post-contrast axial imaging at different times after onset. Second day (A1‐A6); 6th day (B1‐B6); 35th day (C1‐C6); 90th day (D1‐D6); 18th months (E1‐E6); Various forms of enhancement including gyri-like (red arrowhead in E2, E3), patchy (yellow arrowhead in E1, B2, B3) and nodular (blue arrowhead in B3, B5, C4) pattern with meningeal enhanced were displayed at 6th and 35th day. MR images on 90th day showed a decrease in degree and number of the lesions. Patchy hyperintense signals in right temporalis muscle and outer pterygium muscle suggested temporomandibular space infection (red arrow in A1, B1, C1).

**Figure 3.**
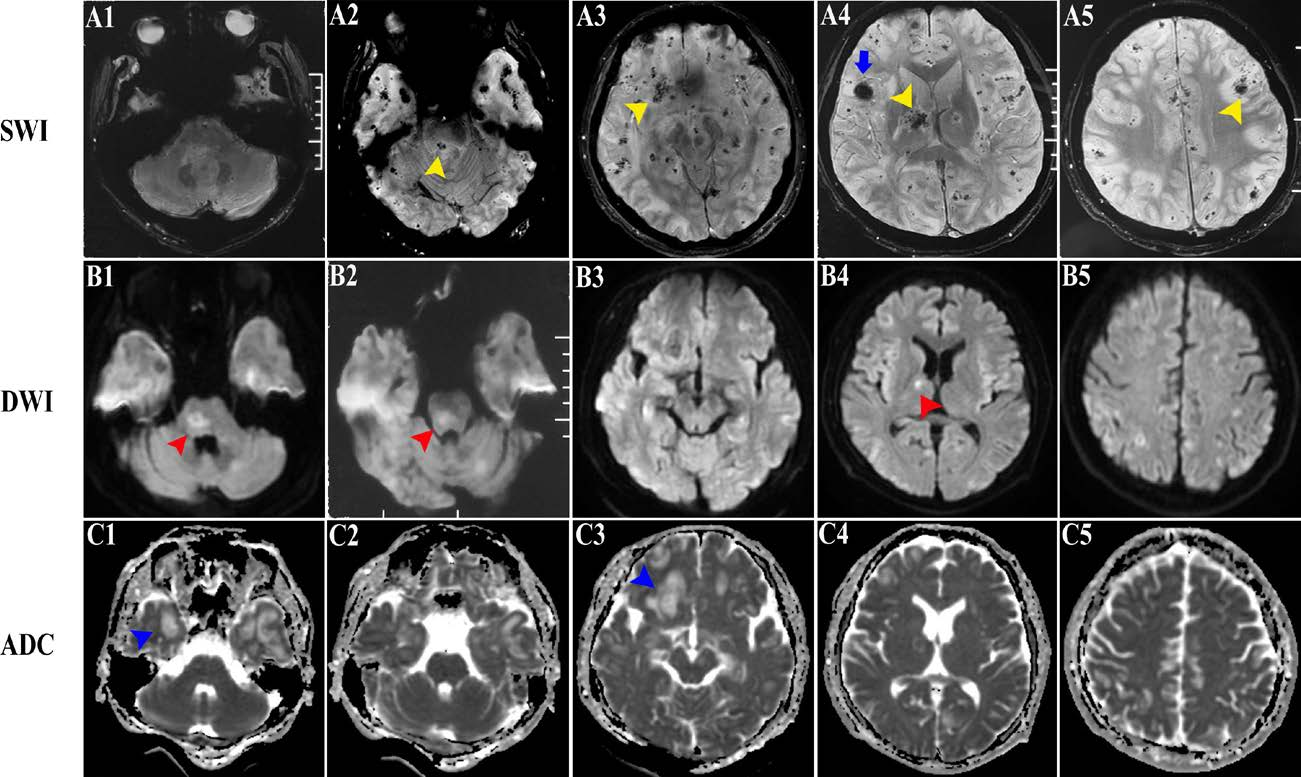
MR images of susceptibility-weighted image (SWI), diffusion-weighted image (DWI) and apparent diffusion coefficient (ADC) map on day 6. SWI present multiple extensive petechial hemorrhage in cortex, white matter, gray-white junction (yellow arrow) and a large intracerebral bleed in right frontal lobe (blue arrowhead). DWI and ADC axial sections reveal diffusion restriction in some lesions (red arrow), but in other lesions apparent diffusion coefficient increases (blue arrow).

**Figure 4.**
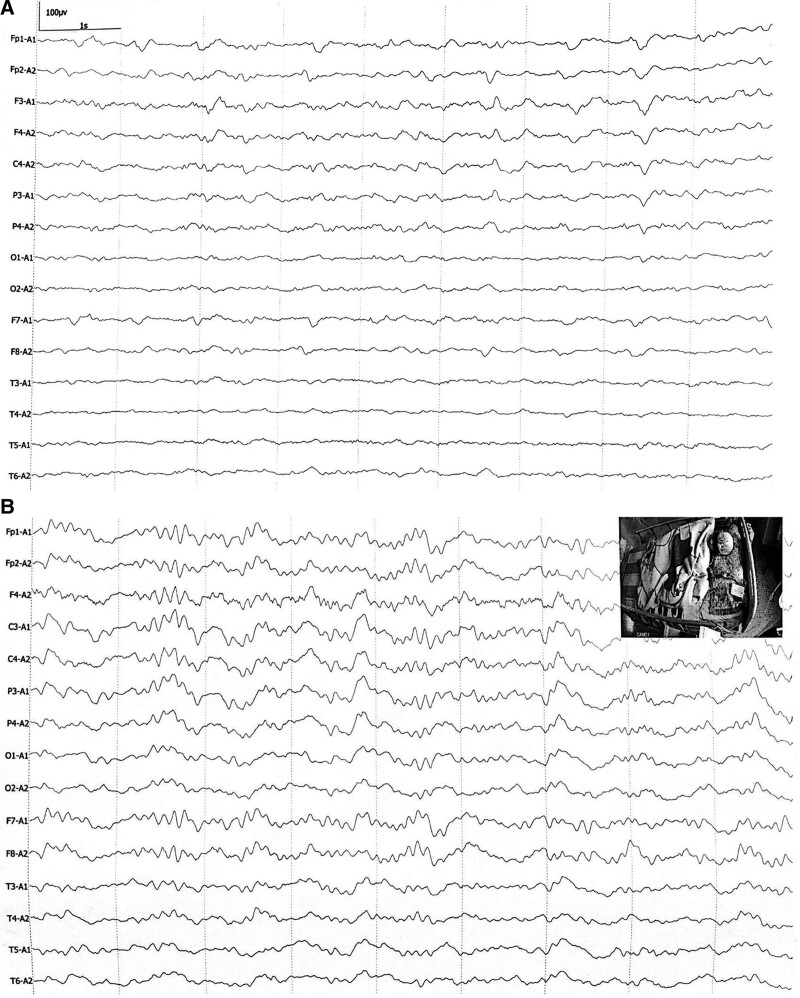
Electroenchephalogram (EEG) excerpt at admission (A) and added treatment with mycophenolate mofetil on day 120 (B). EEG at admission shows slowing of background activity in both hemispheres with epileptiform discharges. EEG on day 120 records sparse spindles mainly in area of anterior head.

He underwent a complete evaluation of infective, metabolic, neuromyelitis optica spectrum disorders, vasculitis profile and autoimmune encephalitis panel. Table 1 shows the pertinent laboratory findings. Inflammatory parameters in the peripheral blood were only mild higher [white blood cell count (WBC) 12.63 × 10^9^ per liter, C-Reactive Protein (CRP, 15.19 mg/L) and erythrocyte sedimentation rate (ESR, 30 mm/h)]. CSF evaluation revealed pleocytosis with a white blood cell count of 15 per microliter of which 60% were polymorphonuclear, red blood cell count of 8 per microliter and increased protein level of 0.53 g/L. Metagenomic sequencing assay for pathogen detection were also performed without positive results. The T2-weighted (T2WI) and fluid-attenuated inversion recovery (FLAIR) images on admission demonstrated plaque-like hyperintense lesion in medulla, bilateral cerebral cortex and white matter (Fig. 1A). A repeat MRI on the sixth day revealed increased number and mass effect of lesions, involving in ponto-medullar region, cerebellum, bilateral frontal lobe, temporal lobe, insula, right thalamus (Fig. 1B). Post-contrast imaging showed gyri-like, patchy and nodular enhancement partially within the regions above (Fig. 2A‐E). Additionally, patchy hyperintense signals with contrast in right temporalis muscle and outer pterygium muscle suggested temporomandibular space infection (Fig. 2A‐C). Some of these lesions presented restricted diffusion particularly in the gray‐white junction, while in other lesions apparent diffusion coefficient increases (Fig. 3B and C). Susceptibility-weighted imaging (SWI) revealed multiple scattered punctate parenchymal hemorrhages and a larger intracerebral bleed, which were strongly suggestive of AHLE (Fig. 3A). Spinal cord MRI was also performed but without any remarkable lesions. Initial EEG showed slowing background activity in both hemispheres with polyspike-waves of frontal predominance (Fig. 4A). Constitutional severity necessitated the patient intensive care. He underwent incubation and tracheostomy soon because of persistent decreasing oxygen saturation. Meanwhile, he received lower intracranial pressure treatment including intravenous hypertonic saline (30 g/L) and mannitol under rigorous monitoring, as well as wide-spectrum probabilistic anti-infectious treatment including meropenem, vancomycin and acyclovir due to aspiration pneumonia and suspected blood-borne disseminated infection. On the tenth day after onset, we administered methylprednisolone intravenously beginning as a dose of one gram per day for 3 days with a tapering dose followed by immunoglobulin at a dose of 30 g over 5 days. The patient did not improve in consciousness though his infectious symptoms were under control. MRI reviewed on day 35 showing a marked progression of white matter lesions and edema in extent (Fig. 1C). His families declined cerebral biopsies and use of plasmapheresis, and persistence of iatrogenic bladder bleeding precluded the use of cyclophosphamide. Thus we instituted mycophenolate mofetil (MMF, 0.75 g, twice a day) and restarted intravenous injection of immunoglobulin (IVIG) at a dose of 0.4 g/kg/day over 5 days every 2 weeks. Sodium valproate was added due to his focal limb seizures several times a day. Rehabilitation exercises at bedside was continued. Oral steroids and IVIG were ceased on day 45 and 60 respectively. On day 85, his brainstem reflexes were improved and he could breathe on his own, so we removed the endotracheal tube later. MRI on day 90 showed mild gyri-like enhancement with enlargement of subarachnoid space (Figs. 1D and 2D**).** On day 120, a bed-side VEEG showed asymmetric, not well-formed spindles at frequency < 10 Hz (Fig. 4B). The patient regained consciousness gradually on day 180 and demonstrated neurologic recovery, manifesting open eyes, staring at an object in direct response to its movement intermittently, and moving spastic extremities spontaneously. MMF was discontinued later due to adverse drug reaction. 12 months later, he displayed subtle facial expression when his families called his name without intelligible verbalization. MRI at the 18th month revealed multiple cerebromalacia foci and partial cerebral atrophy. He had no evidence of expressive language function but demonstrated visual pursuit, which indicated he was in minimally conscious state (MCS). He remained in this state without further improvement until now.

**Table 1:** Laboratory examinations.

Diagnostics	Results
	Pressure: 20 cm H_2_O
	RBC (per microliter): 8
	WBC:15 with polymorphonuclear predominance of 93%
Cerebrospinal fluid studies	Glucose: 3.8 mmol/L
	Total protein: 0.53 g/L
	Culture: No growth
	mGNS: Negative
	Oligoclonal bands: Negative
	Serum WBC (per liter): 12.63 × 10^9^
	CRP (mg/L): 15.19
	ESR (mm/H): 30
	Serum respiratory pathogens IgG and IgM: Negative
	Serum agglutination test of brucellosis: Negative
Systemic infectious work-up	Widal’s reaction: Non-reactive
	T-SPOT.TB: Negative
	Tuberculosis PCR: Negative
	Hepatitis, syphilis and HIV profiles: Negative
	Blood culture: Negative
	Endotracheal tube culture: carbapamenase-positive Klebsiella pneumonia
	Oligoclonal bands: Negative
	mGNS: Negative
	Serum creatinine (umol/L): 108
	GFR (mL/min): 80.30
	Myoglobin (ng/mL): 233
	Liver function test: Normal
Ancillary tests	Thyroid function test: Normal
	Antiganglioside antibodies: Negative
	Autoimmune encephalitis panel test: Negative
	Coagulation function test: Normal
	Antinuclear antibody spectrum: Negative
	Arthritis associated antibodies: Negative
	Antiphospholipid antibodies: Negative

ESR = erythrocyte sedimentation rate, GFR = glomerular filtration rate, mGNS = metagenomics next generation sequencing, PCR = polymerase chain reaction, RBC = red blood cell, TB = tuberculosis, WBC = white blood cell.

## 3. Discussion

AHLE, also termed Weston‐Hurst disease, is a rare and severe variant of acute disseminated encephalomyelitis (ADEM) presenting with rapid neurologic deterioration associated with perivascular hemorrhage and edema. The pathologic hallmark is perivascular demyelination, mixed inflammatory infiltrates, fibrinoid necrosis of the vessels along with hemorrhage in a “ring and ball” pattern.^[8,9]^ In the latest systematic review, an overall mortality of AHLE is 46.5%,^[6]^ which is substantially lower than 70% previously reported. 39.5% survivors remained disabled and only 14% recovered completely.^[10]^ Various pathogens have been postulated as a trigger for AHLE. HSV, VZV, EBV and HHV-6 have been found in brain biopsies of patients. Bacteria like mycoplasma, streptococcus and leptospira have also been reported.^[11,12]^ COVID-associated AHLE cases differ from their counterparts predated the pandemic that the preceding systemic infection was often severe and mortality was common.^[13]^ Multiple cell types and broad cytokine are also involved pathologically. Lori et al^[14]^ described upregulation of C3, MAC and IL-1 in two individuals with AHLE in the context of partial complement factor I (FI) deficiency. New Added treatment with IL-1 receptor antagonist, anakinra led to improvement of clinical status. Michaela et al reported in two AHLE children, T cell-associated cytokine elevation (interleukins 6, 8, and 17A) and higher CCL2 or CCL3 than in patients with ADEM, without B cell-associated cytokine elevation.^[15]^ Autosomal dominant mutations and heterozygous variant identified in *RANBP2* may sensitize the brain to inflammation and predispose to AHLE.^[16]^ Another genetic predisposition for AHLE has been found in patients with particular major histocompatibility complex (MHC) haplotypes.^[17]^ Excessive immunological response which causes demyelination may be due to cross-reactivity between human myelin antigens and viral or bacterial antigens.^[18]^ Pirko et al^[19]^ demonstrated the development of hemorrhagic demyelination in C57BL/6 mice infected with Theiler’s Murine Encephalitis Virus (TMEV) within 24 h through intravenous administration of CD8^+^T cell restricted peptide VP2_121–130_ viral capsid. All above evidence indicate both cellular and humoral immune mechanisms likely contribute to the phenotype, possibly modulated by the nature of preceding infections and host factors (including individual susceptibility and genetics).^[15]^

Despite absence of histopathologic microscopic findings obtained via stereotactic biopsy, characteristic features of our patient supported the diagnosis of AHLE. First, our patient is a male adult person, as 67% male preponderance reiterated in literature, while children and teenagers are more liable to be succumbing to ADEM. Second, antecedent history of infection or vaccination are reported in 50% to 75% of patients and 19% of them are not identified with an underlying pathogen.^[20]^ As in our case, the patient was preceded by overt odontogenic temporomandibular space infection and pneumonia after exodontia, which indicated anaerobe infection may be the trigger. Third, our patient exhibited a hyperacute, monophasic clinical course with rapidly progressive symptoms followed by coma, which is consistent with classical course of AHLE. Fourth, CSF and neuroimaging findings are also compatible with profiles of AHLE. A higher cell count with pleocytosis and presence of red cells, which points towards hemorrhagic conversion of lesions in the brain. AHLE has a variable scope of MRI findings.^[21]^ MRI reveals confluent white matter lesions with significant edema, space-occupying effects and petechial hemorrhages. Unparalleled to previous report that gray matter was relatively spared,^[7,22]^ our case showed multiple cortex involved. Contrast enhancement is inconsistent, depending on the damage to the blood brain barrier in different phases which correlates with pathologic appearance.^[23,24]^ SWI avails to distinguish AHLE from other common demyelinating diseases where the extensive hemorrhage is not characteristic. In ADEM, hemorrhage is generally lacking and lesions are smaller with less prominent cerebral edema. Hung-Wen Kao et al^[25]^ reported that non-displaced medullary veins with concomitant petechial hemorrhage as a unique diagnostic clue of AHLE, excluding the differential diagnosis of malignant tumors which exert mass effect and distort brain parenchyma.

The main principle is attenuating the autoimmune process, avoiding secondary neurological damage and preventing infectious complications. Glucocorticoids were the most common immunosuppressive therapy applied (97%), followed by plasmapheresis (26%), and intravenous immunoglobulins (12%).^[26,27]^ However, response to immunomodulation therapy exhibited variable in AHLE against ADEM, which shows a uniformly good response to these therapies. Unlike classical ADEM, the response to steroid seemed pessimistic and therefore most doctors resorted to PLEX/IVIG immediately.^[5,28]^ In cases of fulminant and refractory ADEM variants, rituximab and/or cyclophosphamide have been reported with some positive results.^[29–31]^ Hsam et al^[33]^ reported the patient treated with complement 5- inhibitor Eculizumab exhibited substantial progress in the power of his limbs when IVMP, PLEX, IVIG and cyclophosphamide were ineffective. These discrepancies may be attributed to the difference in the pathological processes in two diseases. ADEM predominantly presents with inflammatory demyelination whereas additional fibrinoid necrosis and hemorrhage present mainly in AHLE, which leads to tissue destruction. Immunotherapies can curtail inflammatory responses but may not have any effect on the irreparably damaged tissues once it is too late.^[20]^

Our patient commenced high-dose IVMP but failed to terminate his deteriorated condition. So we restarted low doses of IVIG every two weeks up to another three courses combined with MMF. IVIG may block the Fc-receptors on macrophages rendering them less auto-aggressive for cell mediated immune response. MMF is a prodrug of mycophenolic acid (MPA), which depletes guanosine nucleotides preferentially in T and B lymphocytes and inhibits their proliferation, hence it suppresses both cell-mediated immune responses and antibody formation.^[34]^ The notable change is detection of some spindles on EEG on day 120. This element of sleep architecture may serve as an early predictor for behavioral awakening in comatose patients.^[35]^ As expected, our patient got improved slowly in his consciousness subsequently. His visual pursuit and response to his name calling from family members suggested he has been in the transition from coma to MCS. Clinical improvement bespeaks of beneficial role of supplement with IVIG and MMF. Initial pulse of steroid may be a synergistic or stabilizing action. It is disappointing that our patient did not show more clearly-discernible behavioral signs of consciousness after 18 months.

## 4. Conclusion

AHLE presents distinctly from classical ADEM, and the situation may pose a diagnostic challenge. Clinicians should be vigilant in recognizing AHLE because of its rapid clinical deterioration and high mortality. We highlight the critical role of multimodal MRI, particularly SWI in the diagnosis of AHLE if cerebral biopsies are unavailable. Multiple courses of IVIG with MMF may be effective when early single pulse of corticosteroids fails. Individual who survives the initial insult may carry relatively good prognosis.

## Author contributions

**Conceptualization**: Jun Yang, Chen Wu.

**Data curation**: Chen Wu, Weiliang Zhang, Yan Jiao, Minhui Dong, Heng Zhou, Yun Lv.

**Investigation:** Chen Wu, Weiliang Zhang.

**Formal analysis:** Chen Wu, Weiliang Zhang.

**Resources:** Weiliang Zhang.

**Supervision:** Chen Wu, Weiliang Zhang, Yan Jiao, Yun Lv.

**Validation:** Heng Zhou, Minhui Dong, Yan Jiao.

**Writing—original draft:** Chen Wu.

**Writing—review & editing:** Yang Jun.
